# Clinical Characteristics of Patients with Takayasu Arteritis Undergoing Open or Endovascular Operations in China

**DOI:** 10.31083/j.rcm2510373

**Published:** 2024-10-22

**Authors:** Xihao Zhang, Liang Gui, Ruihao Li, Zhiyuan Wu, Zuoguan Chen, Yongpeng Diao, Yuqing Miao, Yongjun Li

**Affiliations:** ^1^Department of Vascular Surgery, Beijing Hospital, National Center of Gerontology, Institute of Geriatric Medicine, Chinese Academy of Medical Sciences, 100005 Beijing, China; ^2^Peking University Fifth School of Clinical Medicine, 100005 Beijing, China; ^3^Department of Vascular Surgery, The First Affiliated Hospital of Nanjing Medical University, 210029 Nanjing, Jiangsu, China; ^4^Peking Union Medical College, Chinese Academy of Medical Science, 100006 Beijing, China

**Keywords:** angiography manifestation, clinical characteristics, surgery, Takayasu arteritis

## Abstract

**Background:**

The operation rate for different involved arteries and the manifestation of vessel involvement of patients with Takayasu arteritis undergoing open or endovascular operations remain unclear. We aimed to investigate the clinical characteristics, vessel involvement, angiographic classification and operations information in a large cohort of patients with Takayasu arteritis undergoing open or endovascular operations at a single centre in China.

**Methods:**

From January 2017 to October 2022, a total of 153 consecutive patients undergoing open or endovascular operations were recruited from the Department of Vascular Surgery of Beijing Hospital. The demographic characteristics, clinical presentations, pattern of vascular involvement and operation information were collected and analysed.

**Results:**

The majority of patients were female (128/153, 83.7%). The most common vascular finding was hypertension (66.7%). The subclavian (74.2%), carotid (70.1%) and renal (68.9%) arteries were the most commonly involved arteries. Type V (40.5%) was the most common angiographic classification pattern. A total of 296 open or endovascular operations were performed, including 73 percutaneous transluminal angioplasties (PTAs), 50 stent placements and 173 bypass graft operations. Patients with renal (83.3%) or carotid (65.2%) artery involvement had markedly higher rates of undergoing operations.

**Conclusions:**

The subclavian and carotid arteries, as well as the type V (40.5%) pattern, exhibited the highest frequency of involvement among patients with Takayasu arteritis who underwent open or endovascular operations. Variations in angiographic features can result in differences in clinical manifestations and significantly impact the possibility and modality of operations.

## 1. Introduction

Takayasu arteritis (TA) is a rare chronic granulomatous vasculitis that mainly involves the aorta and its primary branches [1]. The disease is reported all over the world, but a high incidence and prevalence of TA were found in East Asia and Turkey [2]. Typically, TA predominantly affects young females under forty years of age [3]. In the early phases of TA, nonspecific constitutional symptoms and signs include fever, general fatigue, neck pain, weight loss and headache/dizziness. Subsequently, inflammation and fibrosis of involved vessels progress, leading to segmental stenosis, occlusion, aneurysm, and even ischemia of involved organs [4, 5].

To date, the pathogenesis of TA is not well understood. Numerous studies have demonstrated that the combination of glucocorticoids (GCs) with various immunosuppressants can effectively control inflammation and active disease [2, 6, 7, 8, 9]. Accumulating evidence has also shown the clinical efficacy of biologic agents, including infliximab (IFX), tocilizumab and B-cell depletion, for TA [10, 11, 12]. Unfortunately, despite an appropriate course of immunosuppressive therapy, persistent active diseases remain common [13].

In cases where medical treatment cannot improve the manifestations, open or endovascular operations seem to be the only choice. Multiple studies have shown that these operations not only improve ischemic symptoms caused by cardiovascular malformations (i.e., stenosis or aneurysm) but also may increase long-term survival in patients with TA [14, 15, 16]. However, the operation rate for different involved arteries and the manifestation of vessel involvement of patients with Takayasu arteritis undergoing open or endovascular operations remain unclear. Here, we performed a retrospective study with a large sample size to investigate the clinical features, imaging findings and operation information for Chinese patients with TA undergoing open or endovascular operations, and conducted a comparison of our data with primarily medicine-treated groups reported in existing literature.We present information to determine the involved vessels that are more commonly in need of these operations to better recognize patients who may need a higher level of surveillance in cases where these operations are inevitable.

## 2. Patients and Methods

### 2.1 Patients and Data Collection

Between January 2017 and October 2022, a total of 153 consecutive patients were recruited from the Department of Vascular Surgery of Beijing Hospital. Subjects meeting all of the following criteria were eligible for the study: (1) diagnosed with TA by two expert rheumatologists according to the 1990 American College of Rheumatology (ACR) criteria and (2) met at least one of the following operation indication: (i) severe stenosis (>70%) or occlusion of the aorta and its branches; (ii) resistant renovascular hypertension; (iii) severe aortic coarctation; (iv) progressive aortic aneurysm enlargement with a tendency for dissection or rupture; (v) critical cerebrovascular or coronary artery ischemia.

Subjects with one or more of the following conditions were excluded: (1) comorbidity with other rheumatic diseases or cancer or infectious disease (except tuberculosis); (2) inability to tolerate anaesthesia and trauma due to poor cardiorespiratory function; (3) mental or other cognitive impairment or refusal to cooperate with necessary experiments; and (4) pregnancy.

The demographic characteristics, clinical presentations, laboratory examination, pattern of vascular involvement and operation information were retrospectively collected and analysed. The angiographic involvement pattern was based on criteria established by Hata *et al*. [17], and the angiographicresults were analysed. Each patient underwent at least one blood vessel examination to assess the extent of blood vessel involvement, which includes catheter angiography, computed tomography angiography (CTA) and ultrasound.

We conducted a search on PubMed for articles using the keywords ‘Takayasu’s arteritis’ and ‘cohort study’ to obtain the clinical characteristics of patients with TA in other studies. Given the low incidence of TA, studies including more than 50 patients were included in this research, and all patients involved in these studies fulfilled the 1990 ACR criteria for TA. Comparisons were conducted between the current study and the studies identified through the literature search.

### 2.2 Vascular Involvement Assess

Each patient underwent blood vessel examinations using either catheter angiography, computed tomography angiography (CTA), or ultrasound, at least once, in order to assess the extent of blood vessel involvement. Stenosis was determined by measuring the ratio between the diameter of the narrowest segment and the diameter of a normal segment of the artery. Restenosis was defined as a target artery narrowing of more than 50% in diameter. Occlusion was defined as an absence of flow in the treated segment. The reporting standards of patency status were defined by the Society for Vascular Surgery.

### 2.3 Statistical Analysis

SPSS 20.0 software (SPSS Inc., Chicago, IL, USA) was used to perform all statistical analyses. Continuous variables are presented as median (Q1, Q3). Categorical data are expressed as the number (percentage).

## 3. Results

### 3.1 Demographic Data and Clinical Characteristics

The clinical features are presented in Table 1. The majority of patients were female (128/153, 83.7%). The most common vascular finding was hypertension (66.7%). This could be a result of hypertension failing to be controlled by appropriate medical therapy being the main leading cause of renal vascular operations for patients with TA and a huge portion of operations performed in this study involved the renal arteries. Cardiovascular risk factors such as diabetes or hyperlipidemia were rare in these patients. Dizziness (37.9%) was the most common neurological manifestation, followed by visual disturbances or loss of vision (18.3%) and headache (12.4%). The median erythrocyte sedimentation rate (ESR), C-reactive protein (CRP) level, WBC count, and creatinine (Cr) level were all evaluated (Table 1).

**Table 1.** **Demographic data and clinical features of 153 Chinese patients with TA who underwent open or endovascular operations**.

Clinical characteristic		No. or value	Proportion (%)
Sex			
	Female	128	83.7
	Male	25	16.3
Age (years)		30.3 ± 12.6	
Inflammatory symptoms			
	Fever	4	2.6
	Neck pain	2	1.3
	Fatigue	3	2.0
Vascular findings			
	Hypertension	102	66.7
	Weak pulse	14	9.2
	Upper limb weakness	10	6.5
	Lower limb weakness	12	7.8
Cardiovascular risk factors			
	Diabetes	0	0
	Hyperlipidaemia	7	4.6
	Hyperhomocysteinaemia	7	4.6
Neurological manifestations			
	Dizziness	58	37.9
	Headache	19	12.4
	Visual disturbance or loss	28	18.3
	TIA	4	2.6
	Stroke	10	6.5
History of tuberculosis		16	10.5
Laboratory tests			
	ESR (mm/h)	8.0 (5.0, 13.0) ^a^	
	CRP (mg/dL)	0.3 (0.1, 0.8) ^a^	
	WBC (10^9^/L)	8.8 (6.0, 10.3) ^a^	
	Cr (µmol/L)	59.0 (50.0, 74.0) ^a^	

^a^ median (Q1, Q3). ESR, erythrocyte sedimentation rate; CRP, C-reactive protein; WBC, white blood cell; Cr, creatinine; TA, Takayasu arteritis; TIA, transient ischemic attack.

### 3.2 Vessel Involvement

The details of vessel involvement are shown in Table 2. Among them, the subclavian (74.2%), carotid (70.1%) and renal (68.9%) arteries were the most commonly involved arteries, and their occurrences of involvement were all above 50%. We found another 4 cohort studies from different geographic areas that also described the details of the involved vessels. The details are shown in Table 3 (Ref. [5, 18, 19, 20]). The occurrence rates of carotid artery and subclavian artery involvement were similarly high, but our study had a relatively higher rate of renal artery involvement than the 4 other studies. Obvious differences in occurrence rates were also found in the aorta between the 5 studies.

**Table 2.** **The manifestations of vessels involved in 153 Chinese patients with Takayasu arteritis underwent open or endovascular operations**.

Artery		Patients with imaging, n	Any arterial lesion	Any arterial lesion%	Stenosis%	Occlusion%	Aneurysm%
Carotid artery		127	89	70.1	19.7	48.8	1.6
	Left	127	87	68.5	29.9	38.6	0.0
	Right	127	78	61.4	29.9	29.9	1.6
Innominate artery		122	44	36.1	31.1	4.9	0.0
Subclavian artery		128	95	74.2	25.0	49.2	0.0
	Left	128	91	71.1	30.5	40.6	0.0
	Right	128	61	47.7	21.1	26.6	0.0
Vertebral artery		124	59	47.6	26.6	21.0	0.0
	Left	124	45	36.3	22.6	13.7	0.0
	Right	124	36	29.0	18.5	10.5	0.0
Ascending aorta		98	11	11.2	10.2	0.0	1.0
Aortic arch		118	18	15.3	12.7	0.0	2.5
Abdominal aorta		102	61	59.8	48.0	6.9	4.9
	AA above RA	102	46	45.1	41.2	0.0	3.9
	AA beneath RA	102	34	33.3	25.5	6.9	1.0
Thoracic aorta		108	38	35.2	34.3	0.0	0.9
Renal artery		122	84	68.9	37.7	30.3	0.8
	Left	122	65	53.3	39.3	13.1	0.8
	Right	122	62	50.8	30.3	20.5	0.0
Mesenteric artery		89	37	41.6	29.2	12.4	0.0
	Superior mesenteric artery	85	36	42.4	30.6	11.8	0.0
	Inferior mesenteric artery	67	3	4.5	3.0	1.5	0.0
Iliacfemoral artery		92	13	14.1	7.6	6.5	0.0
	Left	92	11	12.0	6.5	5.4	0.0
	Right	92	12	13.0	7.6	5.4	0.0

RA, renal artery; AA, abdominal aorta.

**Table 3.** **Comparison of involved vessels between the previous series and the present study**.

Artery		This study (n = 153)		Li *et al*. (n = 411) [18]		Lee *et al*. (n = 204) [19]		Mwipatayi *et al*. (n = 272) [20]		Schmidt *et al*. (n = 126) [5]	
		Left	Right	Left	Right	Left	Right	Left	Right	Left	Right
Carotid artery		68.5%	61.4%	79.1%		72.1%	63.7%	30.5%		50.9%	41.7%
	Stenosis	29.9%	29.9%	58.6%		33.3%	32.3%	21.7%		41.7%	37.0%
	Occlusion	38.6%	29.9%	24.8%		21.1%	9.8%			10.2%	5.6%
	Dilatation	ND	ND	3.9%		1.0%	1.5%	ND		ND	ND
	Aneurysm	0	1.4%	0.5%				6.3%		0.9%	0.9%
Innominate artery		36.1%		19.7%		46.8%		10.5%		25.5%	
	Stenosis	31.1%		14.8%		16.3%		8.1%		18.9%	
	Occlusion	4.9%		3.9%		3.0%				6.6%	
	Dilatation	ND		1.9%		4.0%		ND		ND	
	Aneurysm	0		0.2%				5.9%		0.9%	
Subclavian artery		71.1%	47.7%	79.8%		67.1%	55.2%	ND		66.3%	41.0%
	Stenosis	30.5%	21.1%	56.4%		26.0%	24.6%	ND		43.3%	36.2%
	Occlusion	40.6%	26.6%	31.6%		34.8%	14.3%	ND		29.8%	4.8%
	Dilatation	ND	ND	2.7%		0.5%	4.4%	ND		ND	ND
	Aneurysm	0	0	1.2%				ND		0	1.0%
Vertebral artery		36.3%	29.0%	28.7%		ND		ND		18.5%	13.0%
	Stenosis	22.6%	18.5%	20.0%		ND		ND		15.7%	9.3%
	Occlusion	13.7%	10.5%	11.2%		ND		ND		2.8%	3.7%
	Dilatation	ND	ND	2.4%		ND		ND		ND	ND
	Aneurysm	0	0	0.2%		ND		ND		0	0
Ascending aorta		11.2%		9.5%		47.8%		23.9%		9.1%	
	Stenosis	10.2%		0.7%		0		5.5%		2.7%	
	Dilatation	ND		9.0%		25.4%		ND		ND	
	Aneurysm	1.0%		0.5%				15.8%		2.7%	
Aortic arch		15.3%		7.8%		37.9%		33.1%		4.5%	
	Stenosis	12.7%		6.1%		0.5%		16.5%		2.7%	
	Dilatation	ND		1.7%		3.4%		ND		ND	
	Aneurysm	2.5%		0.2%				13.2%		1.8%	
Abdominal aorta		45.1%^a^	33.3%^b^	38.4%		63.2%		68.4%		23.7%^a^	27.4%^b^
	Stenosis	41.2%	25.5%	32.6%		38.3%		42.3%		20.4%	25.3%
	Occlusion	0	6.9%	2.9%		4.0%				1.1%	2.1%
	Dilatation	ND	ND	0		6.0%		ND		ND	ND
	Aneurysm	3.9%	1.0%	1.4%				15.1%		2.2%	1.1%
Thoracic aorta		35.2%		17.5%		57.2%		58.1%		19.1%	
	Stenosis	34.3%		17.5%		22.9%		36.0%		18.2%	
	Occlusion	0		0		0				0	
	Dilatation	ND	ND	3.4%		3.0%		ND		ND	
	Aneurysm	0.9%		0.5%				14.0%		0.9%	
Renal artery		53.3%	50.8%	48.9%		32.2%	31.7%	ND		18.7%	20.9%
	Stenosis	39.3%	30.3%	44.3%		25.2%	25.2%	ND		16.5%	19.8%
	Occlusion	13.1%	20.5%	10.5%		6.0%	4.5%	ND		4.4%	2.2%
	Dilatation	ND	ND	1.5%		0	0	ND		ND	ND
	Aneurysm	0.8	0	0.5%				ND		0	0
Mesenteric artery		42.4%^c^	4.5%^d^	29.7%		22.8%^c^	3.5%^d^	37.1%		24.7%^c^	6.9%^d^
	Stenosis	30.6%	3.0%	21.9%		14.4%	0.5%	33.8%		18.0%	2.3%
	Occlusion	11.8%	1.5%	8.0%		5.9%	2.0%			6.7%	4.6%
	Dilatation	ND	ND	0.2%		1.0%	1.0%	ND		ND	ND
	Aneurysm	0	0	0				3.3%		0	0

^a^Suprarenal aorta. ^b^Infrarenal aorta. ^c^Superior mesenteric artery. ^d^Inferior mesenteric artery. ND, no data.

### 3.3 Numano Classification

Seven studies that also detailed the angiographic involvement pattern based on the classification of Hata *et al*. [17] were selected to compare the angiographic classification between the previous literature and the present study. The details are shown in Table 4 (Ref. [5, 18, 19, 21, 22, 23, 24]). In the 7 studies (including the present study), the type V pattern was the most common and had a similarly high prevalence. However, one study from China showed that type I was the most common pattern [5, 18, 19, 21, 22, 23, 24]. This study had the highest proportion of individuals with Type IV compared to other studies.

**Table 4.** **Comparison of the angiographic classification between the previous series and the present study**.

	This study	He *et al*. [21]	Sun e*t al*. [22]	Danda *et al*. [23]	Chen *et al*. [24]	Li *et al*. [18]	Lee *et al*. [19]	Schmidt *et al*. [5]
Total (*n*)	153	240	81	585	97	411	204	126
Type I (%)	24.2	22.9	11.1	20.9	34.0	22.1	11.1	20
Type IIa (%)	4.6	3.8	8.6	0.5	21.7	3.9	8.6	6
Type IIb (%)	4.6	4.6	6.2	3.6	7.2	3.9	14.1	7
Type III (%)	4.6	2.9	13.6	5.5	2.1	2.9	4.0	5
Type IV (%)	21.6	19.2	9.9	18.3	5.2	6.3	7.6	5
Type V (%)	40.5	46.7	50.6	51.3	29.9	60.8	54.5	57

### 3.4 Open and Endovascular Operations

**Fig. 1.**
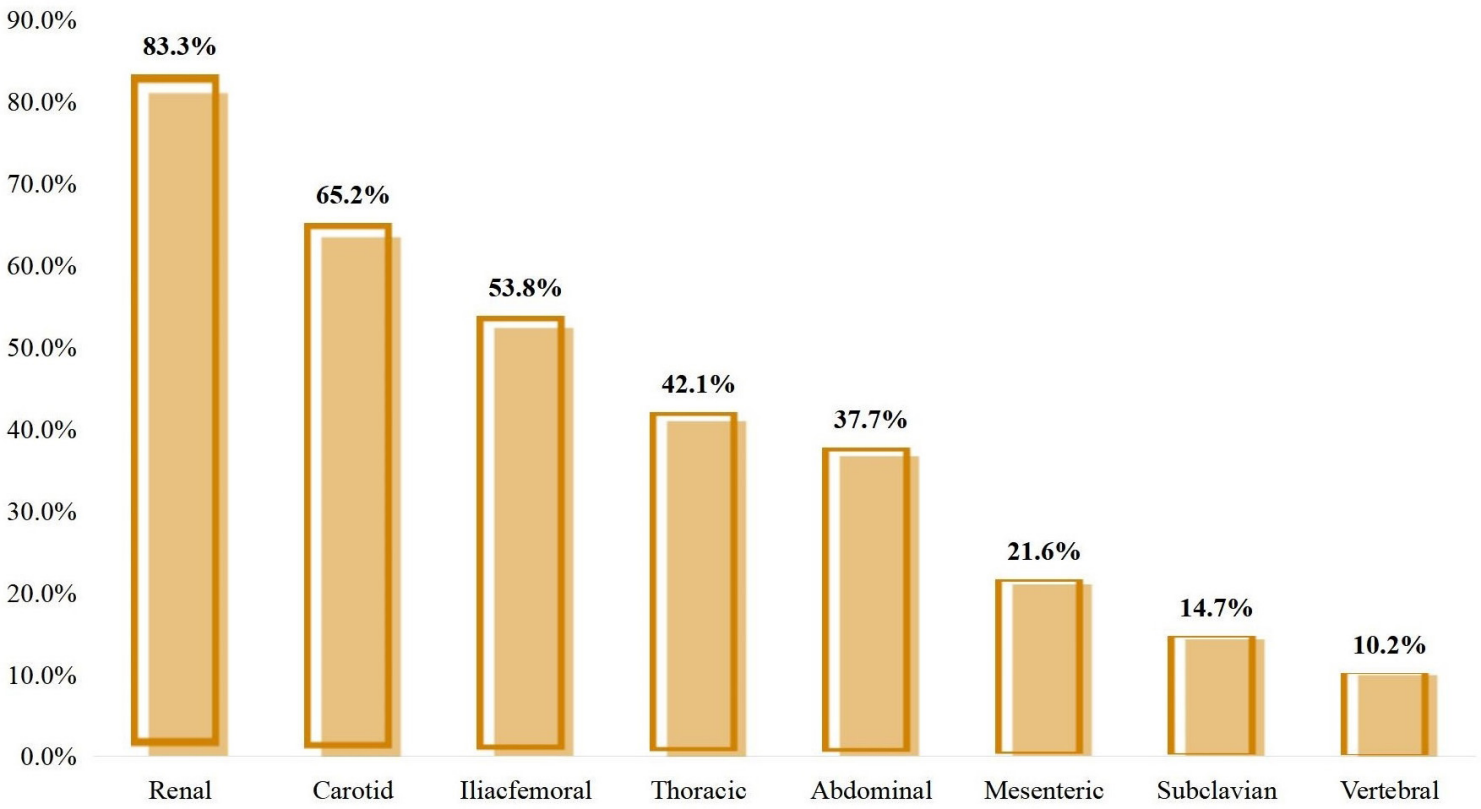
**Operation rate for different involved vessel**.

A total of 296 open or endovascular operations, including 73 percutaneous transluminal angioplasties (PTAs) (24.7%), 50 stent placements (16.9%) and 173 bypass graft operations (58.4%), were performed in 153 patients. The details are shown in Table 5. Operation rates for different involved vessel are ranked and showed in Fig. 1.

**Table 5.** **Open and endovascular operations performed in all 153 Chinese Takayasu patients**.

		PTA	Stent	Bypass	Total	Patients underwent operations	Patients with involvement	Operation rate for involved vessel (%)
Supraaortic branches		20	14	82	116			
	Carotid	13	9	69	91	58	89	65.2
	Vertebral	0	2	6	8	6	59	10.2
	Subclavian	7	3	7	17	14	95	14.7
Aorta		11	18	20	49			
	Thoracic	4	7	10	21	16	38	42.1
	Abdominal	7	11	10	28	23	61	37.7
Renal		39	11	58	108	70	84	83.3
Iliacfemoral		1	4	8	13	7	13	53.8
Mesenteric		2	3	5	10	8	37	21.6

PTA, percutaneous transluminal angioplasty.

Large differences in the number of operations and the rate of individuals undergoing operations were found between the different involved arteries. As shown in Table 2, carotid and renal arteries were the most commonly involved arteries. We found that not only were these two arteries more commonly involved, but they were also more commonly in need of revascularisation operations. There were 84 patients with renal artery involvement in this study, 70 of them underwent operations to revascularise the renal artery. This figure was 58 out of 89 for patients with carotid artery involvement. The operation rates in renal and carotid arteries were 83.3% (70/84) and 65.2% (58/89), respectively. Although subclavian arteries were also commonly involved, only 14 patients underwent operations to revascularise their subclavian arteries. This means only 14.7% of patients with subclavian artery involvement underwent operations in this study. Operation rates for the vertebral artery (10.2%) in patients with vessel involvement were relatively low, while for the thoracic aorta (42.1%) and abdominal aorta (37.7%), they were relatively higher. The modalities of these operations also varied for the different involved arteries. PTAs were more common in renal artery operations (39/108, 36.1%) compared to general (24.7%). Unlike renal artery operations, bypass graft operations (69/91, 75.8%) were more common than general (58.4%) in carotid operations. Compared to operations involving the thoracic aorta, operations involving the abdominal aorta had a higher proportion of endovascular operations.

The vertebral artery (16.7%) was the most common vessel involved when more than one operation was required to revascularise for restenosis, followed by the renal artery (14.3%) and abdominal aorta (13.0%).

## 4. Discussion

In this article, we demonstrate the clinical characteristics, vessel involvement, angiographic classification and operation information in a large cohort of patients with TA undergoing open or endovascular operations at a single centre in China. To the best of our knowledge, this is the largest series of patients with TA who were admitted to a department of vascular surgery requiring open or endovascular operations. Past studies were usually focused on patients who were admitted to a department of rheumatology or a department of cardiology where operations, especially bypass graft operations, were not the main modality of treatment; notably, previous reports have shown that surgical bypass has superior patency to endovascular treatments [25]. Multiple studies have shown that open or endovascular operation can improve ischaemic symptoms and increase long-term survival in patients with TA. Limited studies from vascular surgery departments have failed to show the operation rate and modality for different involved arteries in detail with a large series of patients.

Clinical characteristics in patients with TA may vary on the basis of geographic region. The demographic characteristics of the cohort study described in this article are similar to those previously described in East Asia [18, 19, 26]. Patients were mainly women and relatively young. The most common vascular finding in this study was hypertension (66.7%). This could be a result of hypertension failing to be controlled by appropriate medical therapy being the main leading cause of renal vascular operation for patients with TA.

Through the review of the literature, it was observed that the incidence of involvement of both the carotid arteries and subclavian arteries was consistently high across five studies, including our study [5, 18, 19, 27]. The involved vessels in patients with TA varied by different geographic areas. The rate of vessel involvement in this study was similar to the results of Li’s study [18], which is also based on Chinese patients with TA and has the largest number of patients. Compared to the results of Li’s study [18], patients with TA undergoing operations had a higher rate of renal artery and abdominal aorta involvement.

In this study, bypass graft operations (58.4%) were the most common operation for patients with TA undergoing open or endovascular operations, followed by PTAs (24.7%) and stent placements (16.9%). The rates of surgery were higher in patients with renal artery (83.3%) and carotid artery (65.2%) involvement. We assume this is the result that renal artery and carotid artery involvement can cause obvious clinical symptoms in a relatively early stage and the consequences can be catastrophic. The leading causes for renal artery and carotid artery operations were hypertension not controlled by medical therapy and dizziness, respectively. In contrast, the subclavian (14.7%) and vertebral (10.2%) had much lower rates of operations. We speculate this is because symptoms from the involvement of these arteries are usually more insidious until the lesion is very severe because of better compensation and the consequences are usually milder. This result suggested that when dealing with TA patients with renal artery or carotid artery involvement, doctors should be more cautious and keep the option of operating in mind more. A higher level of surveillance is also needed for these patients because the involvement of these arteries has a much higher possibility of requiring an operation. In the meanwhile, subclavian and vertebral involvement seem less worrying, as they rarely needed operating on.

### Limitations

Our study has several limitations, including its single-centre retrospective design. Another weak point of this study is that in this real-world retrospective study, some important variables that are not routinely collected in usual care were not available, such as functional scores and quality-of-life indicators. Some imaging information was also not available as not all patients had all of the desired examinations.

## 5. Conclusions

In conclusion, the subclavian and carotid arteries, as well as the type V (40.5%) pattern exhibited the highest frequency of involvement among patients with Takayasu arteritis who underwent open or endovascular operations. In this article, patients with renal artery and carotid artery involvement had a much higher rate of undergoing an operation. Variations in angiographic features can result in differences in clinical manifestations and can significantly impact on the possibility and modalities of operating.

## Data Availability

All data points generated or analyzed during this study are included in this article and there are no further underlying data necessary to reproduce the results.

## Abbreviations

TA, Takayasu arteritis; PTA, percutaneous transluminal angioplasty.

## References

[1] Koening CL, Langford CA (2008). Takayasu’s arteritis. Current Treatment Options in Cardiovascular Medicine. *Current Treatment Options in Cardiovascular Medicine*.

[2] Saritas F, Donmez S, Direskeneli H, Pamuk ON (2016). The epidemiology of Takayasu arteritis: a hospital-based study from northwestern part of Turkey. *Rheumatology International*.

[3] Keser G, Direskeneli H, Aksu K (2014). Management of Takayasu arteritis: a systematic review. *Rheumatology*.

[4] Vaideeswar P, Deshpande JR (2013). Pathology of Takayasu arteritis: A brief review. *Annals of Pediatric Cardiology*.

[5] Schmidt J, Kermani TA, Bacani AK, Crowson CS, Cooper LT, Matteson EL (2013). Diagnostic features, treatment, and outcomes of Takayasu arteritis in a US cohort of 126 patients. *Mayo Clinic Proceedings*.

[6] Yoshida M, Watanabe R, Ishii T, Machiyama T, Akita K, Fujita Y (2016). Retrospective analysis of 95 patients with large vessel vasculitis: a single center experience. *International Journal of Rheumatic Diseases*.

[7] Alibaz-Oner F, Aydin SZ, Direskeneli H (2013). Advances in the diagnosis, assessment and outcome of Takayasu’s arteritis. *Clinical Rheumatology*.

[8] Cong XL, Dai SM, Feng X, Wang ZW, Lu QS, Yuan LX (2010). Takayasu’s arteritis: clinical features and outcomes of 125 patients in China. *Clinical Rheumatology*.

[9] de Souza AWS, da Silva MD, Machado LSG, Oliveira ACD, Pinheiro FAG, Sato EI (2012). Short-term effect of leflunomide in patients with Takayasu arteritis: an observational study. *Scandinavian Journal of Rheumatology*.

[10] Serra R, Grande R, Buffone G, Scarcello E, Tripodi F, Rende P (2014). Effects of glucocorticoids and tumor necrosis factor-alpha inhibitors on both clinical and molecular parameters in patients with Takayasu arteritis. *Journal of Pharmacology & Pharmacotherapeutics*.

[11] Mekinian A, Comarmond C, Resche-Rigon M, Mirault T, Kahn JE, Lambert M (2015). Efficacy of Biological-Targeted Treatments in Takayasu Arteritis: Multicenter, Retrospective Study of 49 Patients. *Circulation*.

[12] Youngstein T, Peters JE, Hamdulay SS, Mewar D, Price-Forbes A, Lloyd M (2014). Serial analysis of clinical and imaging indices reveals prolonged efficacy of TNF-α and IL-6 receptor targeted therapies in refractory Takayasu arteritis. *Clinical and Experimental Rheumatology*.

[13] Maz M, Chung SA, Abril A, Langford CA, Gorelik M, Guyatt G (2021). 2021 American College of Rheumatology/Vasculitis Foundation Guideline for the Management of Giant Cell Arteritis and Takayasu Arteritis. *Arthritis & Rheumatology*.

[14] Isobe M (2013). Takayasu arteritis revisited: current diagnosis and treatment. *International Journal of Cardiology*.

[15] Saadoun D, Lambert M, Mirault T, Resche-Rigon M, Koskas F, Cluzel P (2012). Retrospective analysis of surgery versus endovascular intervention in Takayasu arteritis: a multicenter experience. *Circulation*.

[16] Diao Y, Yan S, Premaratne S, Chen Y, Tian X, Chen Z (2020). Surgery and Endovascular Management in Patients With Takayasu’s Arteritis: A Ten-Year Retrospective Study. *Annals of Vascular Surgery*.

[17] Hata A, Noda M, Moriwaki R, Numano F (1996). Angiographic findings of Takayasu arteritis: new classification. *International Journal of Cardiology*.

[18] Li J, Sun F, Chen Z, Yang Y, Zhao J, Li M (2017). The clinical characteristics of Chinese Takayasu’s arteritis patients: a retrospective study of 411 patients over 24 years. *Arthritis Research & Therapy*.

[19] Lee GY, Jang SY, Ko SM, Kim EK, Lee SH, Han H (2012). Cardiovascular manifestations of Takayasu arteritis and their relationship to the disease activity: analysis of 204 Korean patients at a single center. *International Journal of Cardiology*.

[20] Mwipatayi BP, Jeffery PC, Beningfield SJ, Matley PJ, Naidoo NG, Kalla AA (2005). Takayasu arteritis: clinical features and management: report of 272 cases. *ANZ Journal of Surgery*.

[21] He Y, Cheng N, Dang A, Lv N (2019). Association between increased arterial stiffness measured by brachial-ankle pulse wave velocity and cardiovascular events in patients with Takayasu’s arteritis. *Clinical and Experimental Rheumatology*.

[22] Sun Y, Ma L, Chen H, Kong X, Lv P, Dai X (2018). Analysis of predictive factors for treatment resistance and disease relapse in Takayasu’s arteritis. *Clinical Rheumatology*.

[23] Danda D, Goel R, Joseph G, Kumar ST, Nair A, Ravindran R (2021). Clinical course of 602 patients with Takayasu’s arteritis: comparison between Childhood-onset versus adult onset disease. *Rheumatology*.

[24] Chen Z, Hu C, Sun F, Li J, Yang Y, Tian X (2019). Study on the association of serum pentraxin-3 and lysosomal-associated membrane protein-2 levels with disease activity in Chinese Takayasu’s arteritis patients. *Clinical and Experimental Rheumatology*.

[25] Lee GY, Jeon P, Do YS, Sung K, Kim DI, Kim YW (2014). Comparison of outcomes between endovascular treatment and bypass surgery in Takayasu arteritis. *Scandinavian Journal of Rheumatology*.

[26] Yang L, Zhang H, Jiang X, Song L, Qin F, Zou Y (2015). Clinical Features and Outcomes of Takayasu Arteritis with Neurological Symptoms in China: A Retrospective Study. *The Journal of Rheumatology*.

[27] Rimland CA, Quinn KA, Rosenblum JS, Schwartz MN, Bates Gribbons K, Novakovich E (2020). Outcome Measures in Large Vessel Vasculitis: Relationship Between Patient-, Physician-, Imaging-, and Laboratory-Based Assessments. *Arthritis Care & Research*.

